# A Simplified Numerical Approach to Examine the Sensitivity of Two-Electrode Capacitance Sensor Orientation to Capture Different Gas–Liquid Flow Patterns in a Small Circular Pipe

**DOI:** 10.3390/s20174971

**Published:** 2020-09-02

**Authors:** Fayez M. Al-Alweet, Artur J. Jaworski, Yusif A. Alghamdi, Zeyad Almutairi, Jerzy Kołłątaj

**Affiliations:** 1 National Center for Oil and Gas Technology, King Abdulaziz City for Science and Technology, P.O. Box 6086, Riyadh 11442, Saudi Arabia; 2 National Center for Corrosion Technology, King Abdulaziz City for Science and Technology, P.O. Box 6086, Riyadh 11442, Saudi Arabia; 3 School of Computing and Engineering, University of Huddersfield, Huddersfield HD1 3DH, UK; a.jaworski@hud.ac.uk; 4 Sustainable Energy Technologies Center (SET), King Saud University, P.O. Box 800, Riyadh 11421, Saudi Arabia; yalghamdi1@ksu.edu.sa (Y.A.A.); zaalmutairi@ksu.edu.sa (Z.A.); 5 Deanship of Scientific Research (DSR), King Saud University, Riyadh 11421, Saudi Arabia; 6 Mechanical Engineering Department, King Saud University, P.O. Box 800, Riyadh 11421, Saudi Arabia; 7 Department of Electrical Engineering, Białystok Technical University, Wiejska 45D, 15-351 Białystok, Poland; amexinfo@amex.pl

**Keywords:** capacitance sensor, electromagnetic, electrostatic, flow pattern, two-phase flow, gas–liquid, simulation

## Abstract

This work involved the simulation of both a multiphase gas–liquid flow and the electromagnetic field representing a two-electrode capacitance sensor in a circular pipe. The simulation investigates in particular the sensitivity of the sensor orientation around the pipe (i.e., top-to-bottom or side-to-side) that best capture the induced flow patterns. The presented numerical work is a simplified simulation by COMSOL multi-physics which was validated by a systematic and an extensive experimental work, using (a) a specially designed simple capacitance sensor (i.e., concave two electrodes), (b) different gas–liquid superficial velocity combinations, (c) different flow section inclinations, and (d) high-speed camera images. The numerical modelling capacitance values were validated against the experimentally measured values which shows a satisfactory level of agreement with a deviation of less than ±2%. The quantity of finite points was between 280,000 and 340,000, which was influenced by the simulated flow pattern. The simulated cases captured the generated flow patterns and their variation inside the pipe, which was in a good agreement when compared to the experimental work as time-dependent values. It was found that the best orientation for the utilized two-electrode capacitance sensor was the top-to-bottom configuration. This is because the sensor’s electrical field distribution was more sensitive, and capable of detecting a greater range of capacitance values. The sensitivity of the top-to-bottom configuration was 1.25–1.64 times greater than that of the side-to-side configuration. Therefore, for design purposes and performance optimization, it is recommended to use the top-to-bottom configuration.

## 1. Introduction

### 1.1. Sensors in Multiphase Flow

The hydrodynamic variables in a multiphase flow system which involves gas–solids/gas–liquid two-phase flow are generally measured and estimated by the utilization of proper devices and sensors. Different sensors to measure these variables are available and each has its own specific functionality and limitations. Briefly, in gas–solids two-phase flow systems (e.g., fluidized bed reactors), to optimize the system performance and reach a high chemical conversion rate, there are a number of variables that must be determined from the experimental measurements. These variables are the solids holdup (including the resident time), the solids circulation rate, the flow regimes, and the pressure drop. In return, there are invasive and non-invasive measurement methods which utilize the appropriate sensor to determine some of these variables [1]. These methods and sensors are (a) a direct measurement of the solids circulation rate by high-speed camera and the visual observation of an accumulated level in the bed, (b) the utilization of fiber optic probes and fiberglass spirals to estimate the solids’ velocity inside the reactor’s riser [2,3], (c) using the pressure and optical sensors to measure the pressure drop and its corresponding solids’ holdup and circulation rate [4], (d) infrared sensor technique to measure the dense solids concentration [5], (e) particles image velocimetry (PIV) was also used to track the solid particles inside the fluidized beds [6], and (f) a force probe was used to examine the generated bubble characteristics [7]. In addition, others used the statistical parameters (such as the standard deviation and the mean) to analyze the signals that were obtained from the pressure sensors to identify the flow regimes [8]. It was found that most of the sensors mentioned above have high accuracy, however, they necessitate regular maintenance and recalibration and thus can be considered time consuming and expensive (except that of the pressure sensors). Alternatively, the pressure sensor can be used reliably, it is characterized by its simplicity, low cost, and un-tedious calibration process. However, caution must to be taken when estimating the solids’ circulation rate from these sensors, because it was reported that the direct measurement of the solids circulation rate from the pressure sensors usually gives an overestimated value [1,9].

This work was concerned with the performance of the sensors which are usually used in a gas–liquid multiphase flow system. Even though there are very large differences between gas–liquid and gas–solids flow, however, the hydrodynamic variables are relatively similar. These variables in gas–liquid multiphase flow are (a) the liquid/gas holdup, (b) the intermittent flow bubble velocities, (c) the pressure drop, and (d) the generated flow patterns. Thus, the performance of any multiphase system that uses gas–liquid flow as a medium in a circular pipe depend on obtaining these variables before and during the actual operation. The implementation of simple or highly sophisticated sensors made the determination of these variables possible. The name and brief principle of work description of the sensors that are usually used for gas–liquid multiphase flow systems in the industrial and academic research sectors are presented in Table 1. In general, these sensors and measurement devices are (a) high-speed camera [10], (b) photochromic dye activation [11,12], (c) particles image velocimetry (PIV) [13], (d) external microphone sensors (i.e., acoustic techniques) [14], (e) probe-based sensors, such as (i) optical fiber probes [15], (ii) two hot film anemometers [16], (iii) capacitance sensors, and (iv) conductivity and conductance probes (even though in principle the conductivity probes are in fact computing conductance based on the capability of the used medium to conduct electrical current, conversely, the conductance probes are influenced by the geometrical dimensions of the electrodes) [17,18,19,20], (f) tomographic methods including 8–16 electrodes’ capacitance, ionizing radiation (i.e., X-rays and γ-rays), nuclear magnetic resonance, and acoustic (i.e., ultrasound) [21,22,23,24,25,26], (g) static and differential pressure transducers [27].

### 1.2. Two-Electrode Capacitance Sensors in Gas–Liquid Multiphase Flow

Simply, the working principle of any capacitance sensor is that the capacitance output is the result of the variation in the two phases’ concentration as they pass the electrodes domain and its corresponding outcome on the dielectric constant [21]. Capacitance sensors come with a minimum number of two electrodes and up to 16 electrodes [25,28,29]. For constructing tomography images and calculating the liquid holdup with a high accuracy (as presented in Table 1), most of the capacitance sensors that were used had at least eight electrodes or more. However, as stated by Reinecke et al. (1997), Bangliang et al. (2000), and Almutairi et al. (2020), more than two-electrode sensors requires the following supporting equipment; an acquisition system, immediate iteration method, multiplexer, an efficient resistance analyzer (or impedance), and a relatively high-speed processer with a display unit to reconstruct the produced images [28,30,31]. Moreover, apparent disadvantages were raised by many scientific researchers such as the blaring and distorted nature of the reconstructed images, the nonlinearity of the measurements, the severe disturbance of the electromagnetic field due to the length of the connected wires, and this approach is considered complex and expensive, which requires regular calibration [21,32].

This work was mainly concerned with the utilization of a concave two-electrode capacitance sensor in a multiphase gas–liquid system to identify the developed flow patterns. A two-electrode capacitance sensor is normally comprised of an excitation electrode, a detection electrode, and a small gap between these two electrodes which is filled with a thin layer of dielectric material or air. Each electrode holds an equal charge value in comparison to the other, however, with an opposite electrical charge [33]. In general, a two-electrode capacitance sensor comes in many shapes including; concave plates, parallel plates, staggered plates, or a double helix plate [34,35,36]. These two-electrode capacitance sensors are usually utilized to measure the liquid/gas volume fraction in the pipe and to classify the flow patterns [28,32,34,37,38,39,40,41,42]. This type of capacitance is considered a very simple sensor, therefore, several operational disadvantages are expected and observed, including (1) low ability to construct tomography images, (2) nonlinearity of the tomography measurements, and (3) tomography images with low resolution [30,31]. Also, it is characterized by a low accuracy in calculating the gas/liquid holdup, in particular at the chaotic region that is at the post-intermittent flow regimes [36]. In an attempt to increase the sensing ability of the two-electrode capacitance sensor, Tollefesn and Hammer (1998) used the helical electrodes configuration to bend the electromagnetic field of the measurement to increase its sensitivity to calculate both the holdup and differentiate between the flow patterns [43].

Despite the limitations in constructing the tomography images and in calculating the gas/liquid holdup and based on the experimental results by many researchers, the two-electrode capacitance (specially the concave-shaped sensors) can be used reliably in identifying all the well defined flow patterns [28,32,34,37,38,39,40,41]. It was found that extending the data analysis of the capacitance values by means of frequency and statistical analysis can identify the flow patterns with great accuracy [32,34,36,38,40]. This can be done by obtaining the frequency and statistical moments (i.e., the standard deviation, the mean, the power spectral density, the kurtosis, and skewness) of the time-dependent output signal of the capacitance.

The performance of all the aforementioned capacitance sensors (i.e., with two or up to 16 electrodes) are influenced by many factors. These factors are the axial length of the utilized electrodes [44], the capacitance material, the ratio between the length and the diameter, the area between the electrodes, the number of implemented electrodes and the geometrical configuration (including the shape) [34,45], the geometrical structure of the sensor insulation part including its material, the dynamic and kinematic conditions of the used phases in the tested pipe (i.e., density, viscosity, and temperature), and the noise or stray capacitance induced by the length of the wires used to connect the electrodes to the data acquisition systems [46,47]. In addition to all of the above, the two-electrode sensors are also influenced by the orientation of the electrodes (i.e., top-to-bottom or side-to-side). This is due to the small opening sandwiched between the electrodes and the distinct shape/mechanism of each of the produced flow patterns. As demonstrated in this comprehensive review, most of the factors that influence the performance of any capacitance sensor have been well investigated in the literature [30,31,34,37,38,39,40,41,44,48,49,50]. However, the sensitivity of the used sensor (with two electrodes) to the generated flow patterns corresponding to the orientation is not well investigated [51,52].

The principle aim of this study was to use a simplified validated numerical model to conduct further analysis to find the optimum orientation of the utilized sensor in a gas–liquid multiphase flow system. This was achieved by the following research steps; (1) performing a comprehensive data acquisition from a gas–liquid multiphase circular pipe experimental setup with a concave two-electrode sensor, (2) the development of numerical simulations model which represents and approximately reflects the actual flow patterns and the utilized sensor with its corresponding electrostatic field distribution, (3) the validation of the model against the experimental results in terms of the time-dependent analysis of the capacitance values, and (4) selecting the best sensor’s orientation which is characterized by a higher sensitivity to the developed flow patterns.

## 2. Experimental Apparatus and Methodology

**Figure 1:**
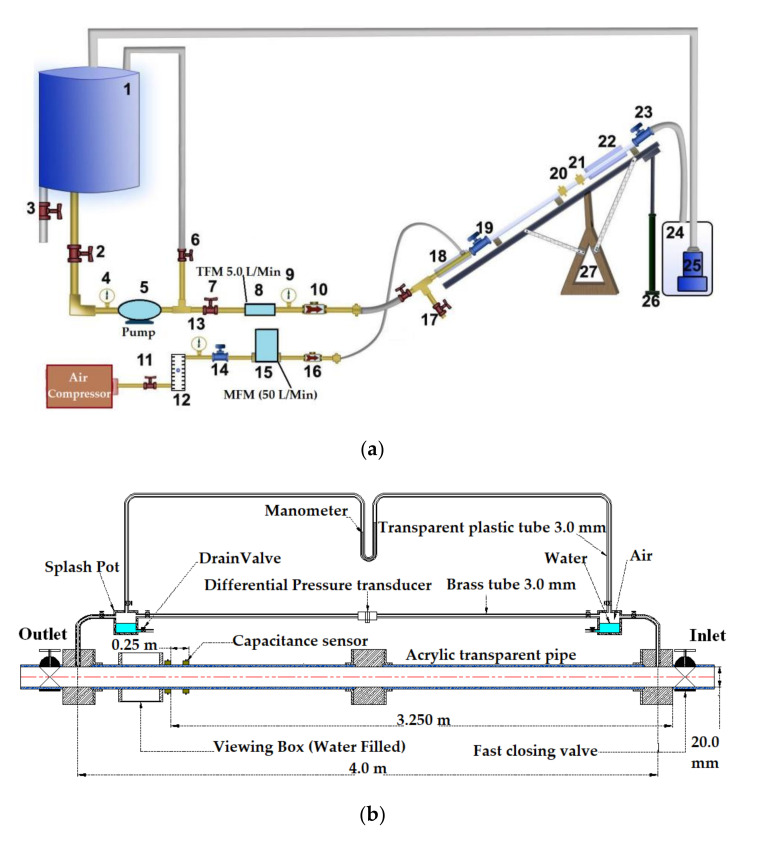

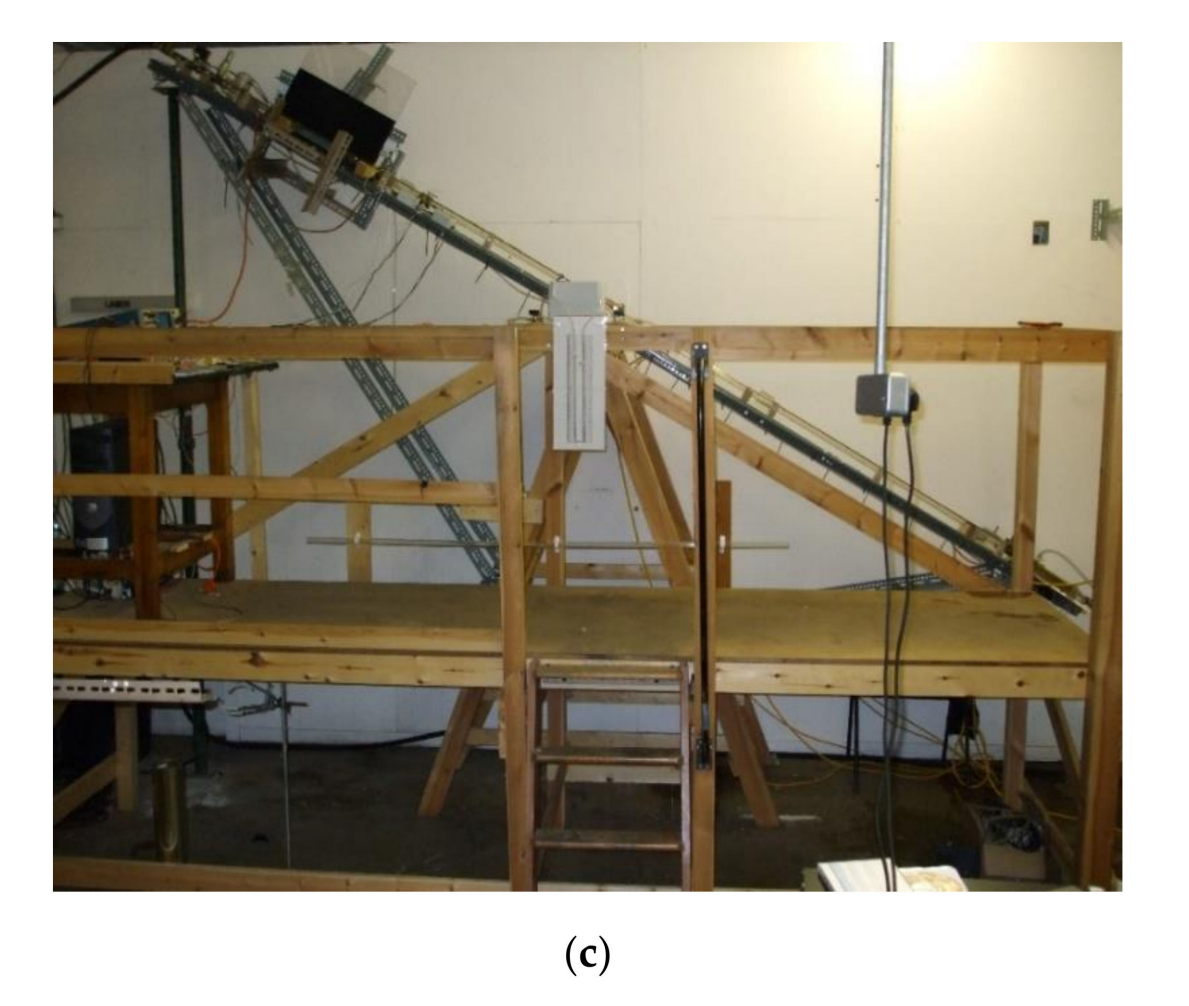
(**a**) Representation of the flow rig: (1) the water tank 1; (2), (6) and (7), the regulator valve; (3) and (17), the drain valves; (4) the temperature gauge; (5) the centrifugal pump; (8) the turbine flow meter; (9) and (13), the pressure gauges; (10) and (16), the check valves; (11) the pressure reducer; (12) the gas ball flow meter; (14), (19) and (23), the fast closing valves; (15) the mass flow meter; (18) the gas–liquid mixer; (20) and (21), the capacitance sensors; (22) the viewing box; (25) the submersible centrifugal pump; (24) the water tank 2; (26) the jack; (27) the swing table; (**b**) the diagram of the test section, and (**c**) the image of the actual experimental setup.

A systematic and comprehensive experimental matrix was developed and conducted on a multiphase gas–liquid apparatus which is shown in Figure 1 (the setup is located at the University of Manchester, UK). The summary of the experimental apparatus specifications and dimensions are presented in Table 2. The experimental operating conditions consist of (a) different combinations of gas–liquid (i.e., air and water) superficial velocities, and (b) different tested inclination angles (i.e., 0°, +15°, +30°). The configuration of this setup and the initial operating conditions allowed the development of all the well defined multiphase gas–liquid flow patterns [28,32,33]. The setup was designed to operate with water as the liquid phase at the superficial velocity between 0 and 1.06 m/s, and with air as the gas phase the superficial velocity is between 0 and 5.0 m/s. The water and air superficial velocities were raised progressively at identical intervals of 0.106 m/s and 0.25 m/s, respectively.

**Figure 2:**
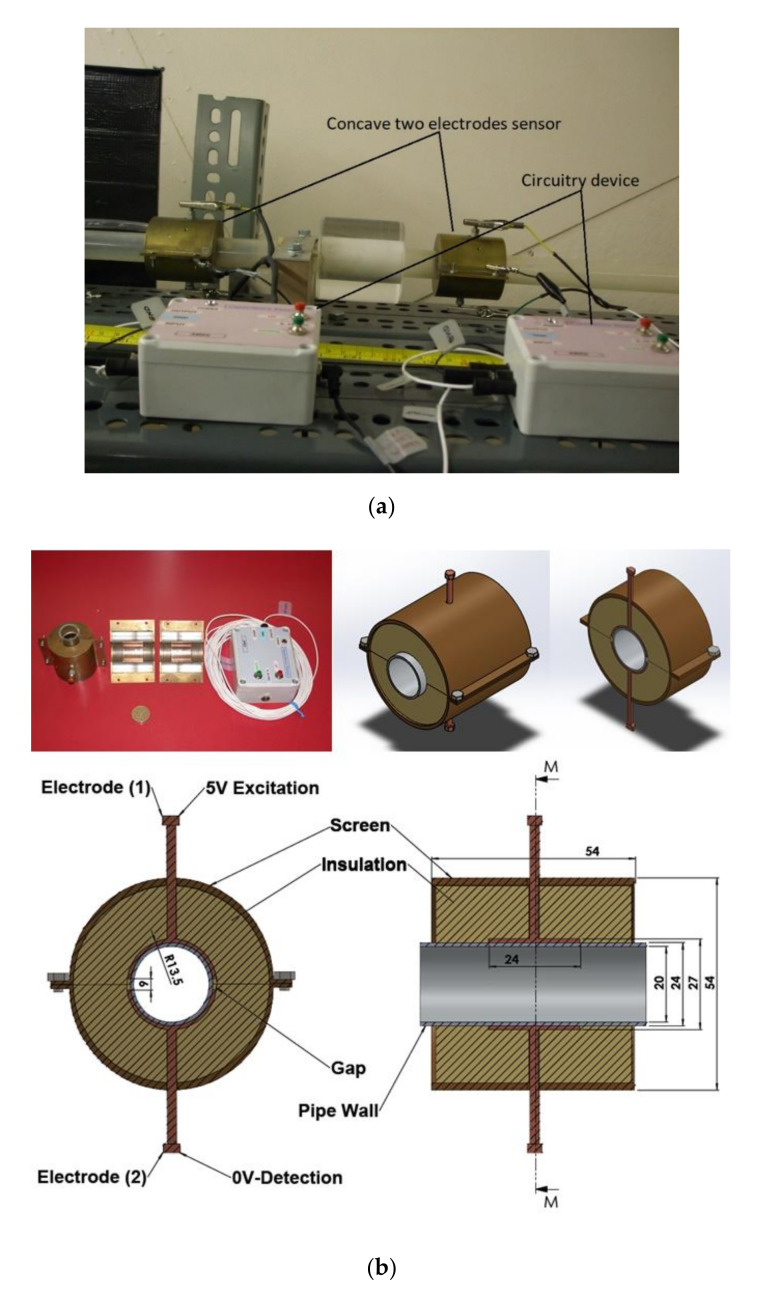
(**a**) The utilized concave two-electrode sensor and the circuitry device, and (**b**) the schematic representation of the concave two-electrode capacitance sensor (dimensions in mm).

Later, a specially designed and manufactured sensor (i.e., concave two-electrode capacitance, Figure 2a) was implemented and validated along with a high-speed camera to capture and identify the generated flow patterns. The physical dimension of the sensors is reported in Table 2 and the schematic representation of the sensor and the electrodes are shown Figure 2b.

In brief, the capacitor is defined as a passive electronic component that holds a charge in the form of an electrostatic field. The concave two-electrode capacitance typically consists of two conducting electrodes (an excitation electrode and a detection electrode) separated by thin layers of dielectric material, such as acrylic or dry air. The two-electrode capacitance sensor electrical design principle, methodology and details are influenced by the research offered by Ferry (1997) and Jerzy (2008) [35,53,54]. More details on the sensor functionality, calibration procedure, responsiveness test, and validation against the experimental measurement are found in Al-Alweet et al. (2020) [32]. The designed sensor captured the generated flow patterns with an accepted accuracy. In addition, the responsiveness of the design sensor to capture the generated flow patterns was very sensitive at low and at high gas–liquid superficial velocities.

As a result of the aforementioned configuration and the operating conditions, comprehensive maps were developed for all the tested inclinations. These maps identify each flow pattern by its visual observed characteristics and the range of gas–liquid superficial gas velocities (The operating procedure shown in more detail here: [32]). The summary of the developed maps is presented in Table 3. The overlap between the two phases’ velocities represents the transitions between the flow patterns and its intersected boundary. Such experimental configuration and equipment resulted on the development of a novel technique that distinguishes each flow pattern based on performing an additional analysis on the frequency and statistical moments obtained from the capacitance values [32].

## 3. Numerical Approach

**Figure 3:**
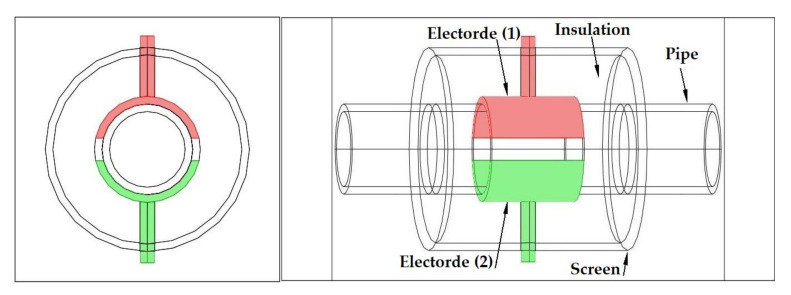
The configuration of the simulated concave two-electrode sensor and the pipe.

As discussed in the introduction section, there are a number of factors influencing the performance of any capacitance sensor including (a) the capacitance-sensing ability for different flow regimes, (b) the shape and configuration of the sensor, (c) the electrode number, length and thickness (also the ratio between the length and the diameter), (d) the orientation of the electrodes around the pipe, and (e) the permittivity of the insulating layer. Thus, it is always a good approach to investigate the effect of one of these factors using numerical simulation. In this work, the focus is on finding the optimum orientation (i.e., side-to-side or top-to-bottom) of the sensor which will help to conduct a full design analysis by obtaining the electrical field distribution and the capacitance values inside the simulated pipe. A simple simulation approach in COMSOL multi-physics was utilized to simulate (a) the typical flow patterns developed inside a circular pipe of a multiphase gas–liquid flow, and (b) the actual sensor and its geometrical model (which is similar to the actual experimental designed capacitance sensor, [32]). Figure 3 shows the computational domain of 12 × 7 × 7 cm, an arrangement defined to reflect the geometrical configuration of the sensor and the pipe. It is worth noting that to increase the sensitivity of the sensor, the pipe outer diameter ratio (i.e., *d*_out_ = 24 mm) to the length of the sensor in the direction of the flow (i.e., axial direction) was 1, in both the actual and simulated sensors. The actual dimensions of the sensor and the pipe in the numerical model and the experiment are briefed in Table 2.

By utilizing the built-in Poisson’s equation available in the COMSOL electrostatics solver [55], the values of the electrical field distribution and the capacitance were simulated and obtained. The three-dimensional form of the equation can be written as follows:

(1) $$\nabla •\left[\varepsilon_{0}\varepsilon \left(x,y,z\right)\nabla V\left(x,y,z\right)\right]=-\rho \left(x,y,z\right)$$

Here, ∇•,∇, $\varepsilon_{0}$, $\varepsilon_{r}^{}\left(x,y,z\right)$, $V\left(x,y,z\right)$ and $\rho \left(x,y,z\right)$ are the divergence operator, the gradient operator, the permittivity of free space 8.85 × 10^−12^ Fm^−1^, the permittivity distribution, the electric potential distribution and the external charge density, respectively. The electric potential V is associated to the electrical field Ε, as specified by Reitz et al. (1993) and Jaworski and Bolton (2000) [29,56,57], and it is stated in the following equation:

(2) $$Ε\left(x,y,z\right)=-\nabla V\left(x,y,z\right)$$

Then, the electric potential $V\left(x,y,z\right)$ in Equation (1) is substituted by the first part of Equation (2), at zero charge density $\rho \left(x,y,z\right)$ in the closed surface. Thus, Equation (1) becomes Laplace’s equation, which reads:

(3) $$\nabla •\left[\varepsilon_{0}\varepsilon \left(x,y,z\right)\nabla Ε\left(x,y,z\right)\right]=0$$

The finite element method in COMSOL is used to solve Equation (3). The surface charge density can then be computed ([56,57]) as follows:

(4) $$Q=\oint_{S}D•nda$$

Here, the symbol D is the electric displacement (vector), *da* is a tiny area on the closed surface *S*, and n denotes a unit vector normal to *da*. The surface charge density can be predicted by the integration performed over the boundary of the sensor electrodes in the COMSOL model where the value of the capacitance is calculated from:

(5) $$C=\frac{Q}{\Delta V}$$

Here, ΔV is the utilized two electrodes voltage difference.

The experimentally used pipe dielectric permittivity was taken into account. According to the manufacturer specifications, the pipe is assumed to have the relative dielectric permittivity of 2.8. The relative dielectric permittivity of air and water was considered to be 1.0 and 80, respectively. The water phase was treated to be as a perfect dielectric involving no conductive influence. The value 80 of the permittivity corresponds to the room temperature condition at which the experiments were operated. In the actual experimental sensor, an acrylic insulating material was used to fill the space between the electrodes and the shield. This material permittivity was considered in the simulation, which was about 2.55.

**Figure 4:**
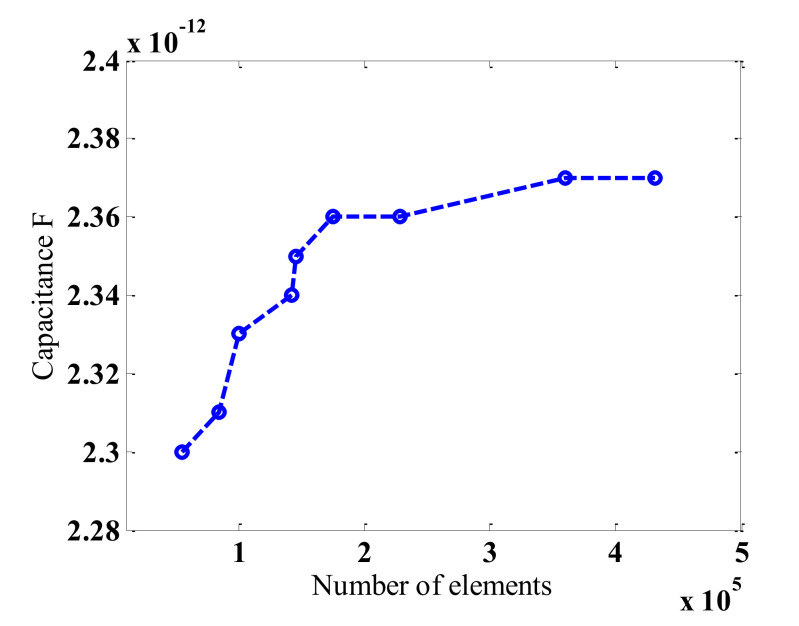
The number of finite points versus the measured capacitance value.

The boundary conditions in the model were defined as follows: (i) the computational domain edge is zero charge/symmetry (n • D = 0), (ii) continuity (n • (D_1_ − D_2_) = 0) for the interfaces between the dielectric materials, (iii) 5 V electric potential is defined for the excitation electrode boundary, and (iv) 0 V electric potential for the detection electrode and the brass shield (screen) boundary. It is very critical to obtain the sufficient number of nodes that allows for an accurate estimation of the capacitance values. Different initial tests were conducted with a variety of numerical solution node numbers that reflect the grid independence of the performed tests. The range as shown in Figure 4 was varied between 50,000 and 420,000. It was found that a plateau behavior was reached between 200,000 and 420,000 nodes. It is very clear now that this range produced no major changes in the capacitance values. Generally, the number of nodes sufficient for an accurate capacitance prediction was between 280,000 and 360,000 nodes.

To be able to simulate two-phase flow patterns, these typical patterns were simplified, as shown in Table 4. The reason behind such simplification is that it is not simple to mimic the exact complex phenomenon of two phases. This is because the patterns mechanism and its corresponding structure changes as a function time. Therefore, the flow patterns simplification involved dividing the volume of the passing medium through the capacitance into an equal interval length of 2 mm. Each interval denotes to the simulated capacitance at a displacement distance of *x* = 2 mm of the simplified model of flow pattern inside the model of the capacitance sensor. In this way, the capacitance of the simulated model for one interval is calculated in a step-by-step manner until the complete flow pattern passed the simulated capacitance sensor for a particular flow pattern. To compare between the simulation and the experimental results, the capacitance fluctuation must be compared as a function of time. Therefore, the investigated flow pattern for a particular simulated case was assumed to pass the simulated capacitance sensor a number of times repetitively (Table 4). This time, for a particular simulated case it was considered to be similar to the time passing in the actual experiment, whereas a similar number of repetitions of the flow pattern passed through the capacitance sensor. To validate the numerical approach used in this work, selected samples of the time-dependent values of the simulated cases and the experimental values were compared (which will be shown and discussed in the next section). The discussion of all the performed experimental flow patterns that are reported in Table 3 and its hydrodynamic characteristics are found in more detail in Al-Alweet et al. (2020) [32]. Again, this work was focused on the selection of the optimum orientation of the designed concave two-electrode capacitance sensor through a numerical approach.

**Figure 5:**
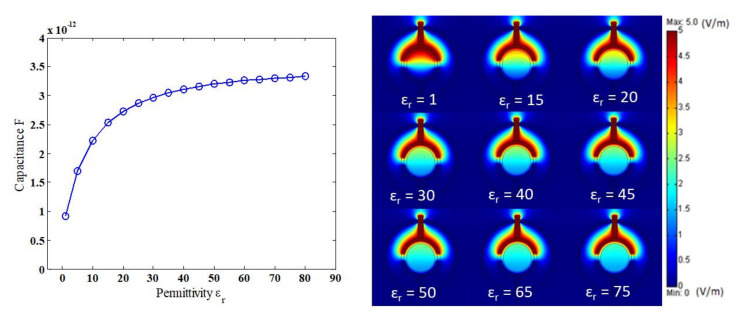
The effect of the permittivity on the simulated sensor capacitance value (ɛ_r_ = 1 for gas phase only and ɛ_r_ = 80 for the liquid phase only).

Initial tests were performed on the numerically modelled sensor to investigate its ability and responsiveness to differentiate between different permittivity (ɛ_r_) and its corresponding capacitance values. For these initial tests, no particular flow pattern was tested, and a homogenous flow was assumed. The permittivity was increased gradually with an equal interval of ɛ_r_ = 5 between ɛ_r_ = 1 up and ɛ_r_ = 80, where 1 corresponds to a gas-phase only, 40 corresponds to a homogeneous gas–liquid mixture, and 80 corresponds to a liquid-phase only. It is worth noting that in the actual experimental work, a homogenous flow cannot be reached with a changing permittivity, only two almost homogenous flows can be achieved, which are gas-phase only (ɛ_r_ = 1) and liquid-phase only (ɛ_r_ = 80). As shown in Figure 5, a sharp increase as the fraction of the gas phase was decreased between 1 and 40, then the sensitivity of the sensor was reduced and steady as the permittivity approached the values between 40 and 80. This is because the pipe wall played an important role in insulating between the two electrodes and the induced permittivity inside the test pipe [29]. Jawroski and Bolton (2000) showed that the relation between the capacitance and the material permittivity in terms of linearity was influenced by the pipe’s wall thickness. The capacitance values of only the gas phase and only the liquid phase were almost similar to the experimental values of the same conditions. In the simulation case, the capacitance for only the gas phase was 0.927 pF, which is similar to the experimental value of 0.926 pF. Similarly, for the only liquid phase, the values between the simulation and experimental were 3.34 pF and 3.33 pF, respectively [32]. It should be emphasized that the unit of the contour line in Figure 5 is in voltage per meter (V/m) because it represents the electrical field. The electrical field between the opposite electrodes is stronger when the permittivity of the medium inside the pipe is high, and it becomes weaker when the permittivity is low. In Figure 5, the contour line of the electrical field shows the distribution of voltage between the excitation electrode and the detection electrode.

**Figure 6:**
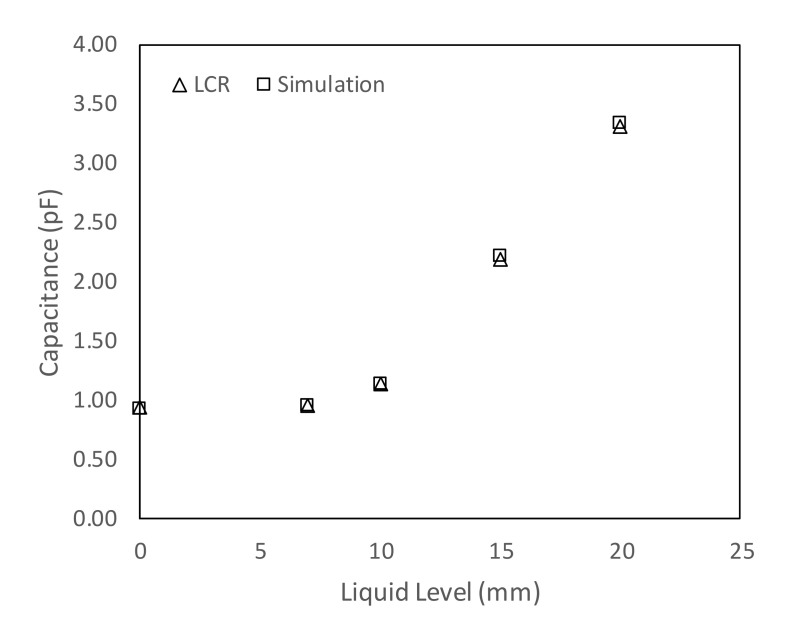
Numerical simulation of the capacitance values vs. LCR capacitance readings.

The numerical model was validated experimentally by two methods, (a) by using a 10 cm test pipe (i.e., closed at both ends at a horizontal inclination), where the water was inserted using a needle from two holes located at the top of the pipe, and (b) by the time-dependent analysis of the simulated capacitance values as the typical well defined multiphase flow patterns were developed. In the first approach, the elevation of water was raised from 0 to 20 mm, and the capacitance readings were taken using the 3532-50 LCR HiTESTER (i.e., inductance (L), capacitance (C), and resistance (R) measurement equipment). The capacitance values obtained from the LCR HiTESTER were then compared with the numerical simulations under the same condition. Figure 6 shows an acceptable level of agreement between the simulated and experimental results. The second approach will be discussed in more detail in the results section.

**Figure 7:**
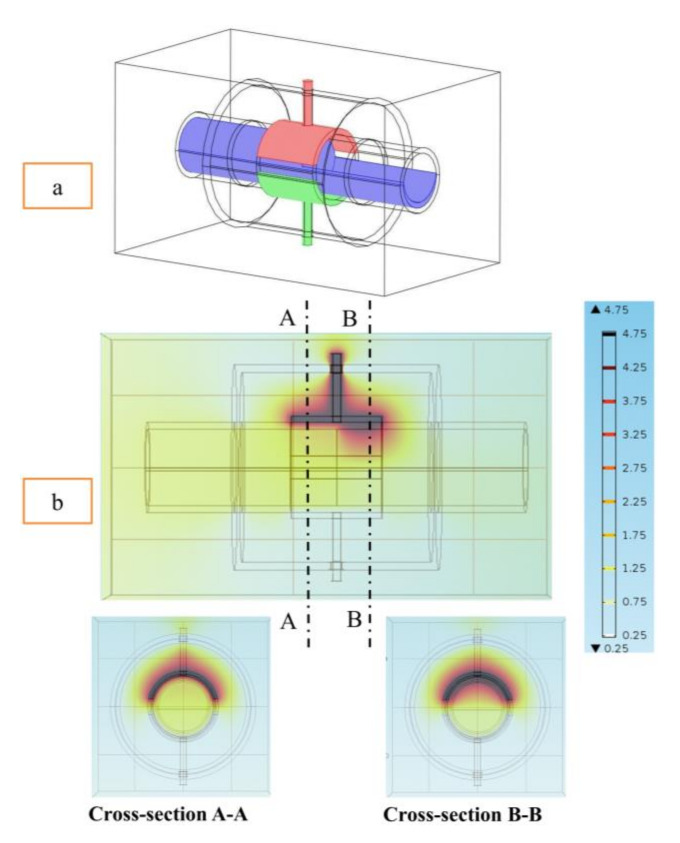
(**a**) Simulated slug flow patterns were investigated numerically using the COMSOL program, and (**b**) the slug flow pattern’s electrical potential distribution in the simulated capacitance sensor (the unit of the contour line is V/m).

The central notion for the aforementioned numerical discussion and the use of the capacitance sensor method in this work was mainly to relate the permittivity distribution of two dielectric media (air and water) in a pipe to the measured capacitance values between the two electrodes positioned around its external circumference. The permittivity distribution in the water–air mixture changed due to the flow pattern crossing through the capacitance sensor, triggering a corresponding change in its measured capacitance. For example, the liquid slug flow pattern was defined as shown in Figure 7a. The front edge of the liquid slug lies between the electrodes. This case was thought to be numerically difficult because of the strong field distortions at all gas/liquid interfaces. The electrical potential distribution (voltage) for the liquid slug is shown in Figure 7b. Laplace’s equation was used to estimate the value of the capacitance for the liquid slug flow pattern for each step as it passes through the sensor. Then, the surface charge density was integrated over the boundary of the detection electrode giving the total charge value for each step. This value is divided by the voltage difference to yield the value of capacitance and thus, the capacitance fluctuation is obtained. For this flow pattern, the average value of the simulated capacitance was 2.35 pF, and for the experimental work was 2.45 pF.

In general, in addition to the above discussion, the following assumptions were taken into consideration for the presented simplified numerical model:

(a) For all the simulated flow patterns, the flow was advancing by 2 mm step until the entire structure of the flow pattern passed the capacitance sensor;
(b) The period of time for the simulation was then taken as identical to the time elapsing in the real experiment while the same number of iterations of the flow pattern crossed the capacitance sensor;
(c) In the small bubble flow pattern, the average sizes of the largest small bubbles and the smallest small bubbles were taken from the high-speed camera images;
(d) The plug and elongated bubble flow patterns have the same hydrodynamic mechanism, however, the sizes of the bubbles are different;
(e) The slug and slug–churn flow patterns have the same hydrodynamic mechanism, however, the slug–churn is frothier (this was implemented by having changing permittivity);
(f) For the annular flow pattern, the pipe wall was assumed to be wetted by a symmetrical liquid film of a thickness of 1 mm over the entire length of the model, except for the section before the capacitance sensor screen, where the thickness of the film was 3 mm, as the simulation was run, this thicker film advanced through the capacitance sensor in 2 mm steps until it filled the entire length of the model. The model assumed that the thickness of the liquid would be symmetric around the pipe;
(g) Regardless of the inclination, the numerical model treated each flow pattern similarly for all inclinations. In other words, for example, the simulated small-bubbles/slug flow pattern is the same in structure for all inclination. The only two differences are (1) the combination of gas–liquid superficial velocities at which these flow patterns were induced due to the effect of gravity and inclination, and (2) some flow patterns did not form or develop in a certain inclination (i.e., the plug flow pattern at a horizontal 0°, and the stratified wavy at all upward inclined angles [28,32]).

## 4. Results and Discussion

### 4.1. Time Dependent Analysis

A validation analysis was carried out by comparing the simulated sensor and its equivalent that is the designed capacitance sensor. This comparison was in terms of the time-dependent output of the capacitance sensor for selected samples of some of the generated flow pattern. It is worth noting that it is very difficult to simulate the time-dependency of the actual experimental work, this is because of the inherent instability of the flow patterns. Thus, as stated in the numerical section, the time for a particular simulated case was assumed to be similar to the time passing in the real experiment. Also, throughout all the experiments, high-speed camera images were captured for all the developed flow patterns. The categorization of all flow patterns by the recorded images was possible, which clearly showed the dispersion of gas and liquid phases within the pipe. The measured capacitance values as a function of time reflected a correct and descriptive visualization of the produced flow patterns when compared to the captured images and their scientific definitions.

#### 4.1.1. Small Bubble Flow Pattern

**Figure 8:**
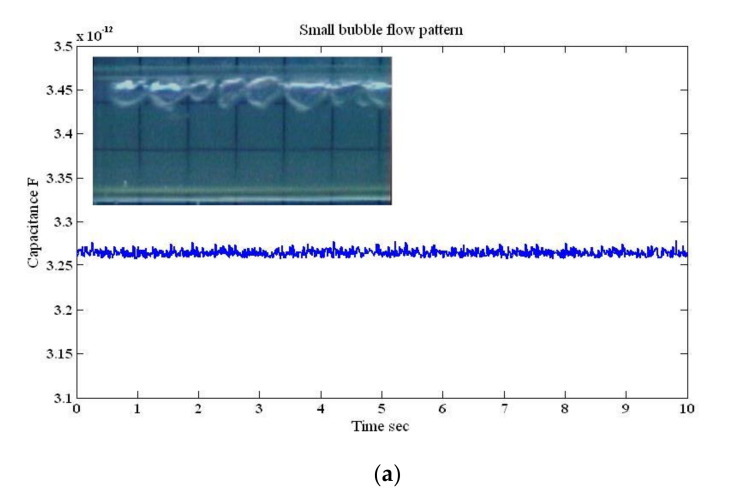

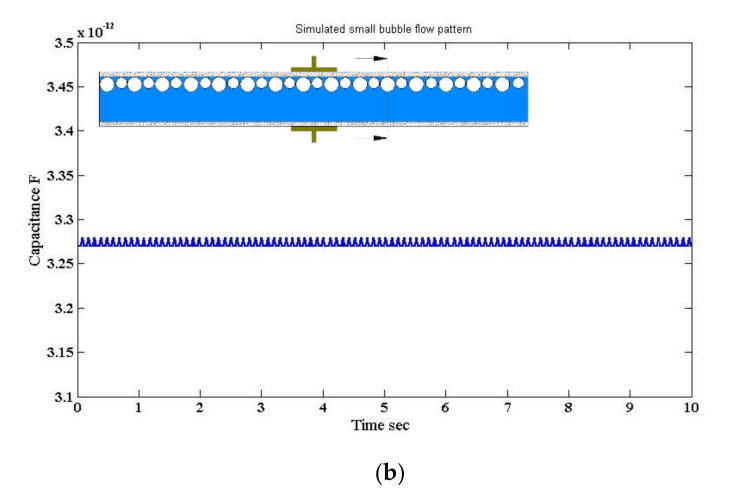
The time-dependent fluctuation of the capacitance value for a small bubble flow pattern: (**a**) experimental [32], and (**b**) simulation (for the superficial gas–liquid phase velocities 0.05 m/s and 0.94 m/s, respectively; inclination angle *β* = 0°).

In the actual experimental work, the small air bubbles (i.e., the gas phase) had a lower liquid holdup and was located at the top section of the pipe, while the water (i.e., the liquid phase) had a higher liquid holdup and occupied a large percentage of the pipe volume. The observed small fluctuation shown in Figure 8a represents the existence of a gas phase at a low holdup. The differences between the only liquid test and small bubbles was very minor, due to the fact that the air here had lower dielectric permittivity than that of the water [32]. For this simulation case, the small bubbles (i.e., small spheres) were assumed to pass the sensor in 10 s as stated in the corresponding flow pattern in Table 2 (i.e., small bubble flow pattern). This was done by converting the step-by-step motion of the spheres into a time domain by assuming that these small bubbles crossed the capacitance 100 times. Figure 8a,b show the comparison between the experimental and the simulation for a horizontal case at the gas–liquid superficial velocity of 0.05 m/s and 0.94 m/s, respectively. The variation in the capacitance values in terms of the time-dependent output was almost similar which indicates good agreement between the experiments (i.e., the designed sensor) and the simulated capacitance traces. The values of the capacitance were 3.26 pF and 3.27 pF for the experimental and simulation cases, respectively.

#### 4.1.2. Plug and Elongated Flow Patterns

**Figure 9:**
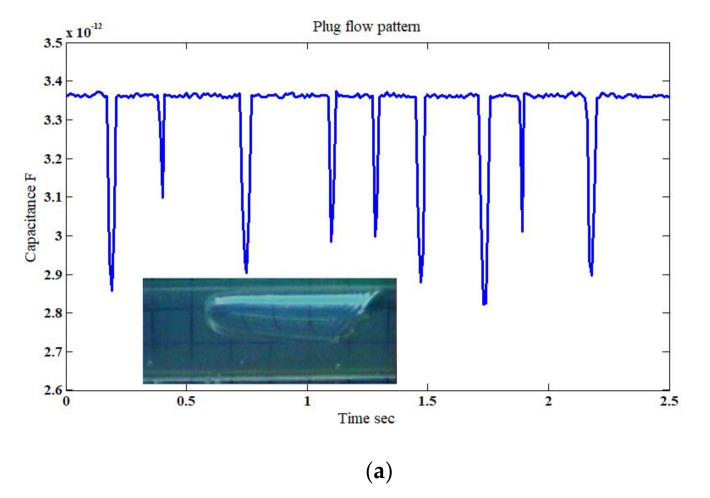

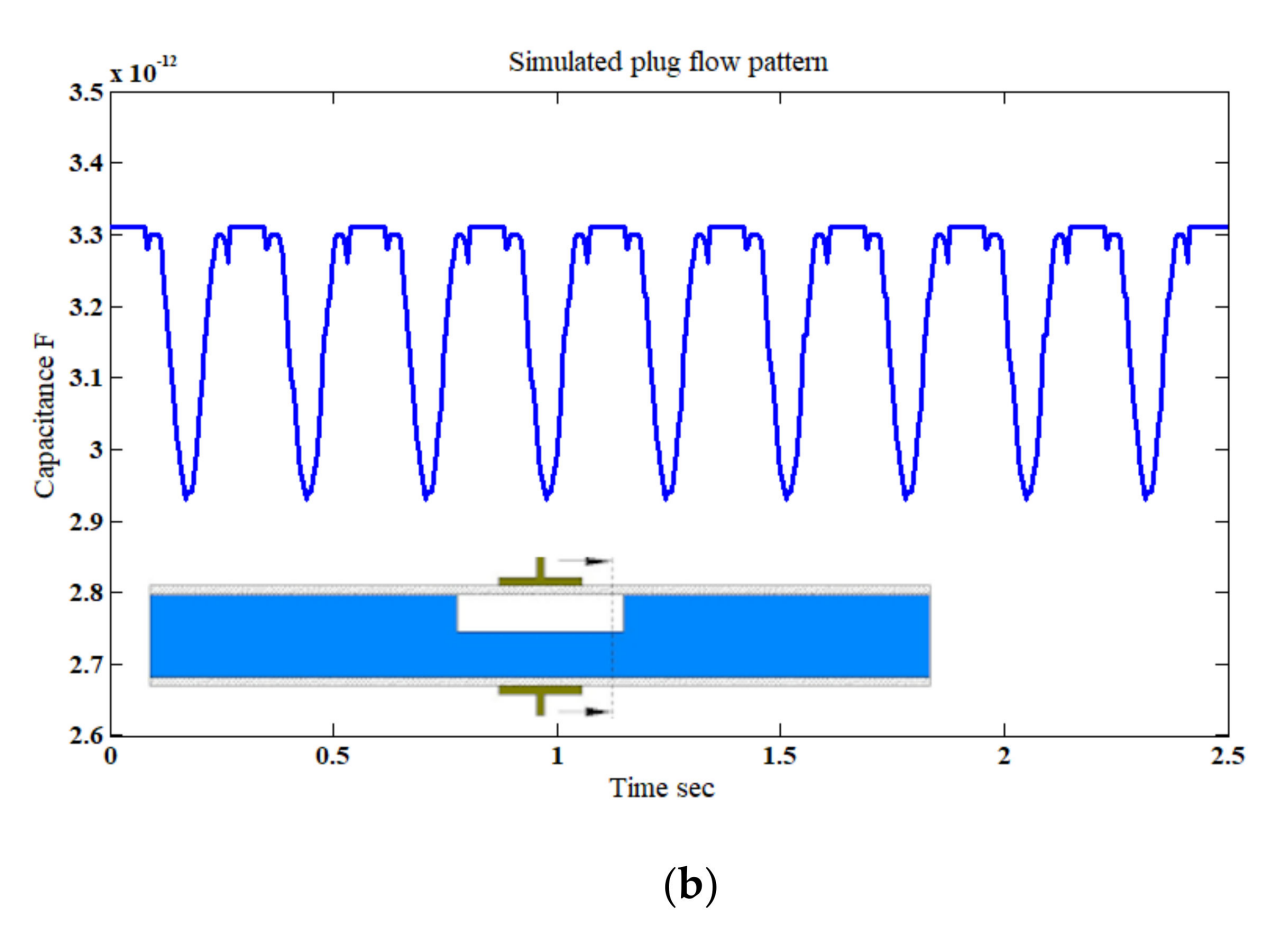
The time-dependent fluctuation of the capacitance value for the plug flow pattern: (**a**) experimental [32], and (**b**) simulation (for the superficial gas–liquid phase velocities 0.212 m/s and 0.26 m/s, respectively; inclination angle *β* = 30°).

The plug flow pattern is generated as a result of relatively increasing the superficial gas velocity (*u*_GS_ = 0.212 m/s) which in return caused the small bubbles to merge together. As shown in Figure 9a for the inclination of +30° and a superficial liquid velocity *u*_LS_ = 0.26 m/s, there is an interchanging behavior between the liquid phase represented by the high capacitance value and the gas phase represented by low capacitance value. Similar behavior was also found in the simulated case, where close agreement was obtained against the experimental results. The capacitance values were in the range of 2.80–3.37 pF and 2.93–3.3 pF for the experimental and the simulation results, respectively, in Figure 9a,b. However, in the actual experiment, the capacitance trace was influenced by the irregular radius and shape of the plug that passed the capacitance sensor, thus it shows as a fluctuation in the minimum values of the capacitance. It is also notable that the slight fluctuation at the maximum capacitance values of the experimental results (Figure 9a) is the artefact of the existence of unmerged small bubbles, but in the simulated case, only one size bubble existed.

The elongated flow bubbles were in principle comparable to that of the plug flow, however, the size of the gas bubble was larger. In the simulation case, the size of the bubble (i.e., cylindrical in shape) was about two times that of the plug flow, as shown Table 4 (estimated by post-processing the images from the experimental work). The experimental capacitance traces were between 2.40 and 3.37 pF and for the simulated traces were between 2.60 and 3.34 pF. However, again in the experimental side, the minimum values of the capacitance varied owing to the irregular nature in the shape of the bubbles, which was similar to that perceived in the plug flow time-dependent values.

#### 4.1.3. Slug and Slug–Churn Flow Patterns

**Figure 10:**
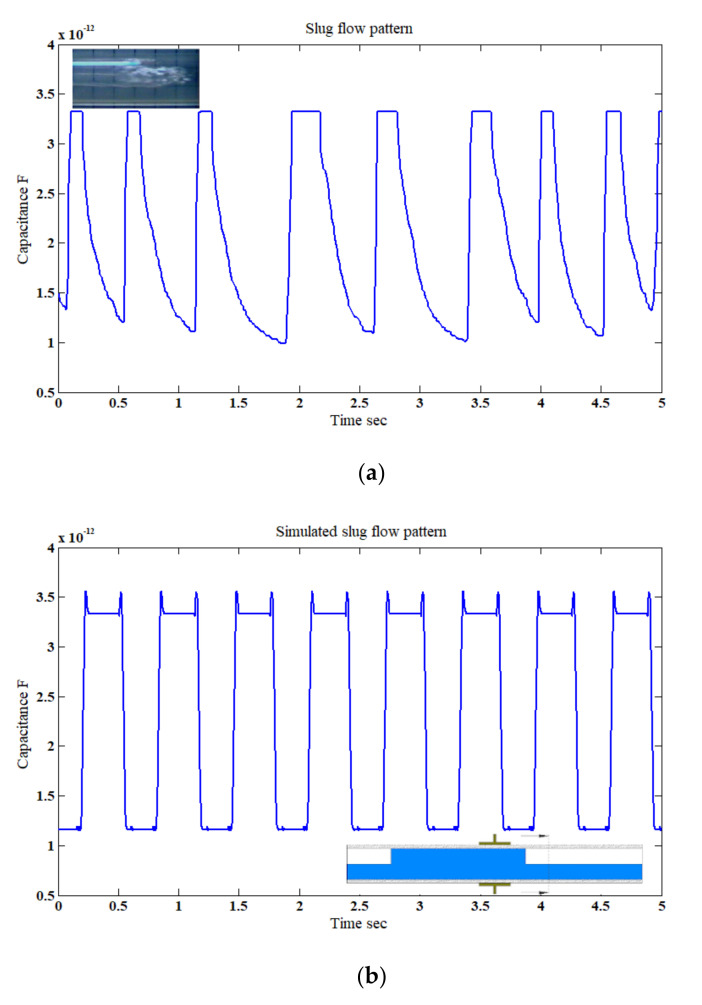
The time-dependent fluctuation of the capacitance value for the slug flow pattern: (**a**) experimental [32], and (**b**) simulation (for the superficial gas–liquid phase velocities of 0.8 m/s and 0.75 m/s, respectively; inclination angle *β* = 0°).

**Figure 11:**
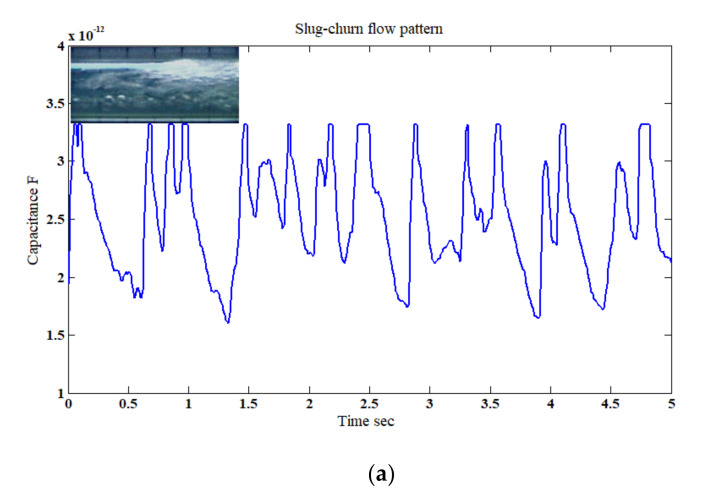

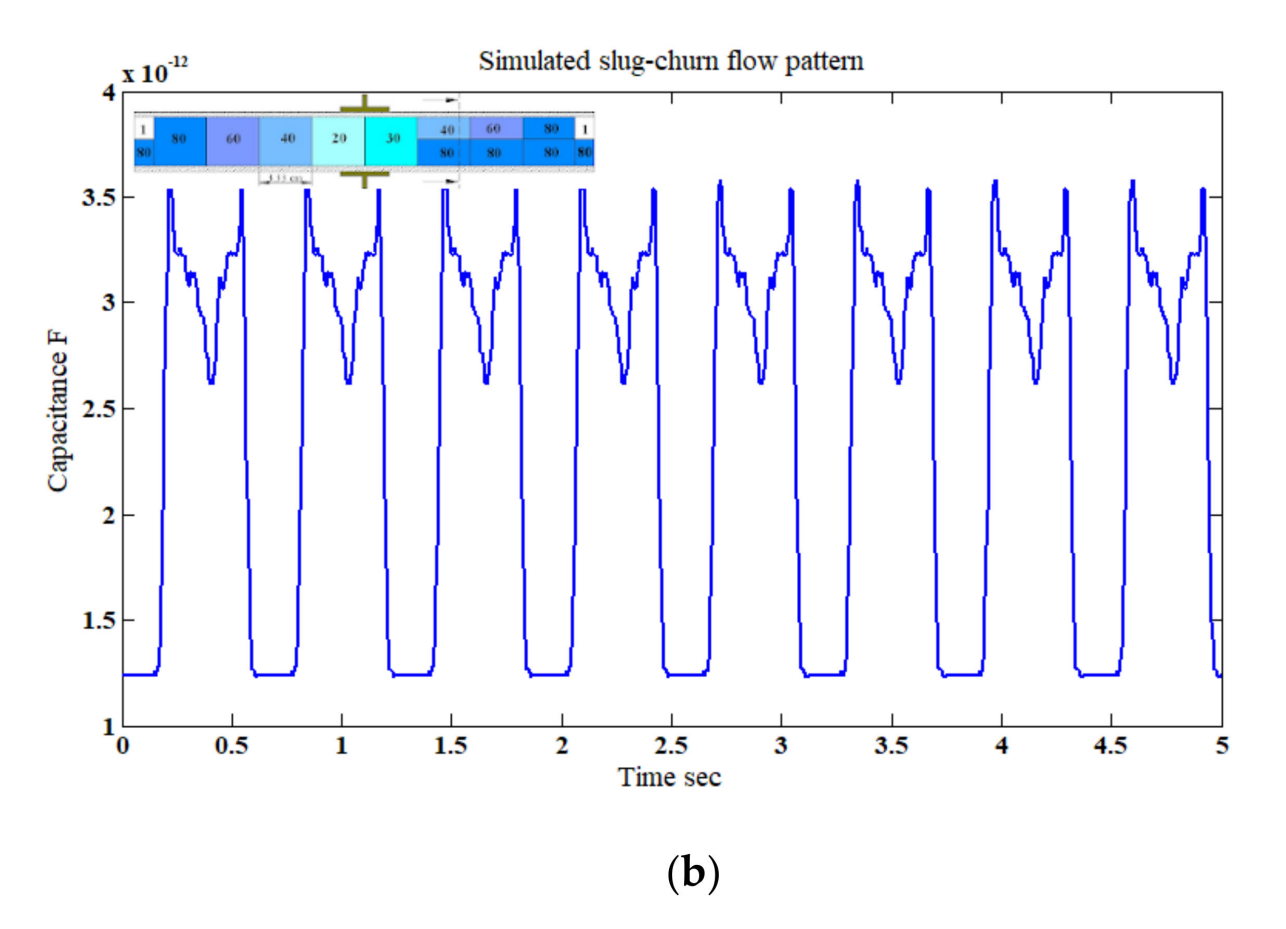
The time-dependent fluctuation of the capacitance value for the slug–churn flow pattern: (**a**) experimental [32], and (**b**) simulation (for the superficial gas–liquid phase velocities of 3 m/s and 0.75 m/s, respectively; inclination angle *β* = 0°).

**Figure A1:**
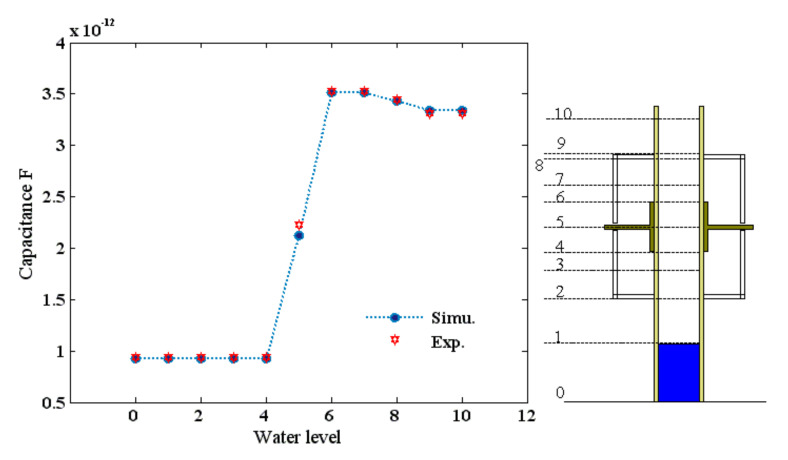
Vertical pipe test to compare between the simulated and experimental capacitance values as a function of changing the elevation of the water inside the pipe.

For the slug flow pattern with *u*_GS_ = 0.80 m/s and *u*_LS_ = 0.75 m/s at a horizontal inclination, different permittivity mixtures of air and water were passing the capacitance sensor intermittently. As shown in Figure 10a,b, the deviation between the mixture of the two phases in the axial direction manifested itself as a different capacitance value. The values for the experimental work varied between 0.96 and 3.33 pF, and for the simulation work with good agreement between 1.2 and 3.34 pF. Similarly, for the slug–churn flow which was generated due the development from the slug flow pattern, for *u*_GS_ = 3 m/s and *u*_LS_ = 0.75 m/s at a horizontal inclination. To be able to simulate the slug–churn flow pattern, the total characteristic length of the slug–churn pattern was estimated experimentally to be approximately 26.66 cm, which was divided into eight sections, each section was 3.33 cm in length and had its own permittivity (Table 4). The fluctuation between the gas and liquid phases caused the capacitance values to vary as a function of time between 1.5 and 3.40 pF and 1.23.40 pF for the experimental and simulation work, respectively. As the relationship between the gas–liquid superficial velocity (*u*_GS_/*u*_LS_) increased (i.e., from 1.1 to 4) the hydrodynamic behavior of the slug flow become slightly bubbly. This is because of the increase in the superficial gas velocity relative to the superficial liquid velocity. Consequently, the capacitance variation was more intense in the slug–churn flow than that of the slug flow pattern, as shown in Figure 11a,b. It is important to state that the spikes observed in Figure 10b and Figure 11b were the outcomes of the shape of the simulated flow patterns which was cylindrical. It is believed that these spikes were caused by the edge of the simulated flow, where a sudden jump in the measured capacitance value was registered at the moment that the edge of the liquid phase entered the sensor’s domain. The rationale of such behavior was discussed in more detail through experimental and numerical validation (as shown in Figure A1) in Appendix A.

#### 4.1.4. Stratified Wavy and Annular Flow Patterns

**Figure 12:**
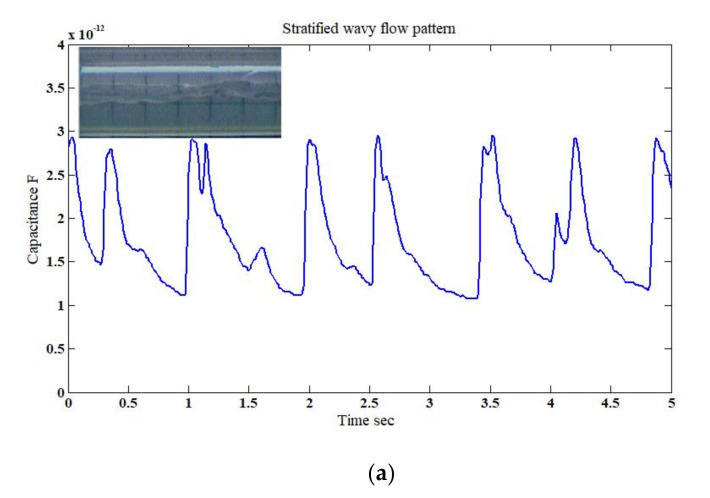

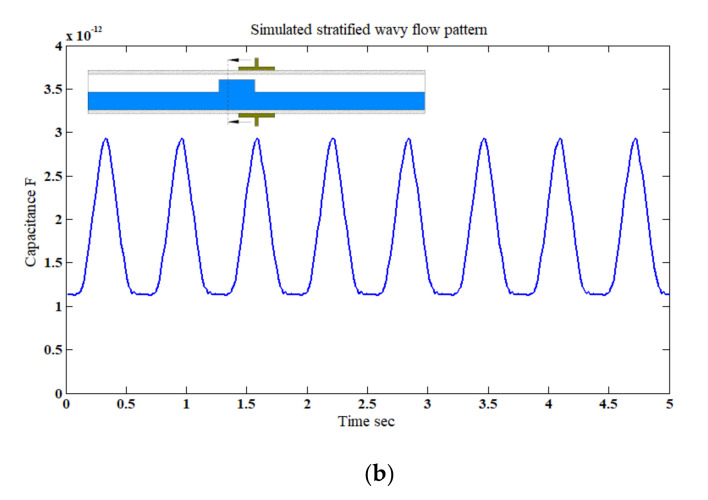
The time-dependent fluctuation of the capacitance value for the stratified flow pattern: (**a**) experimental [32], and (**b**) simulation (for the superficial gas–liquid phase velocities of 3 m/s and 0.318 m/s, respectively; inclination angle *β* = 0°).

In general, a stratified wavy flow pattern has three different and distinct characteristics which are (a) air (i.e., the gas phase) positioned at the upper section of the pipe, (b) water (i.e., the liquid phase) positioned at the bottom half of the pipe, and (c) a small wavy behavior which represents a slight mixing between the two phases. The values of the capacitance sensor for stratified wavy flow for the experimental work and simulation were very close and about 1.1 pF and 3.0 pF for the minimum and maximum values, respectively. The capacitance values measured by the designed sensor where in good agreement with the numerical results as shown in Figure 12a,b. The observed differences in the capacitance fluctuation trend between them was the artefact of the smoothness of the liquid phase in the simulated stratified flow followed by a discrete wave of constant shape. While in the actual case, the stratified wavy flow structure as a function of time was irregular.

**Figure 13:**
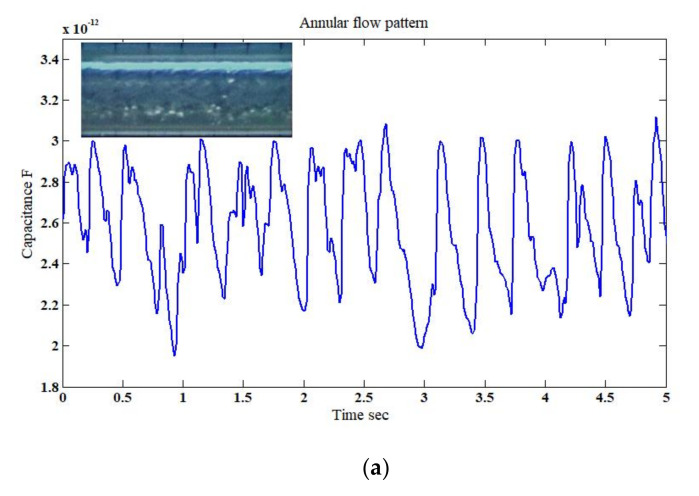

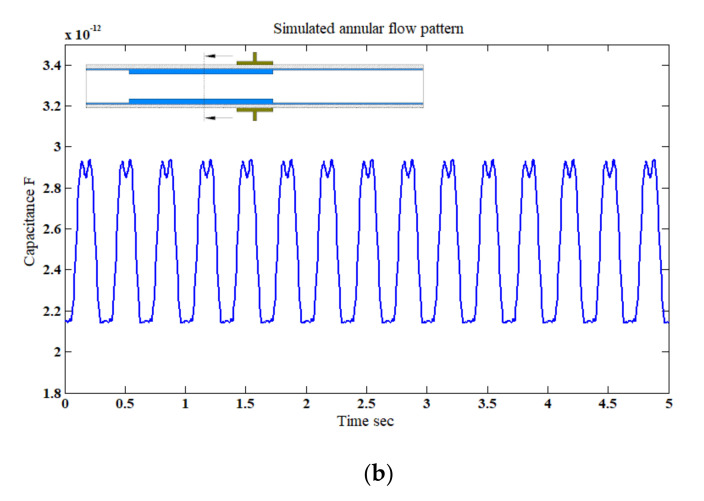
The time-dependent fluctuation of the capacitance value for an annular flow pattern: (**a**) experimental [32], and (**b**) simulation (for the superficial gas–liquid phase velocities of 5 m/s and 1.06 m/s, respectively; inclination angle *β* = 30°).

For the annular flow, the air filled the core of the un-uniformed thickness of the annular film of the water which was adjacent to the pipe wall. With such un-uniformed waves continuously flowing about the pipe boundary wall, the produced border (i.e., interface) between the two phases caused the capacitance sensor to fluctuate, as shown in Figure 13a,b. In the actual experiment, the annular flow pattern was characterized by a fluctuating water film at the annuals of the pipe and air at the core. In the simulation case, it was assumed that there was (i) a wet pipe at the annuals with 1 mm liquid thickness (i.e., symmetrical film), (ii) a wave represented by a 3 mm liquid film (increased in a step-by-step manner by 2 mm as the flow passes the capacitance) which corresponds to the irregularity of the annular flow on the actual experiment, and (iii) a gas phase at the pipe’s core. Good agreement between the experimental work and simulation is evident, and the capacitance values fluctuated between 1.90–3.10 pF and 2.10–2.90 pF, respectively. It was observed that the minimum value in such a flow pattern when compared to slug and slug–churn flow patterns was doubled to about 2 pF. This behavior could be attributed to the hydrodynamic mechanism/structure of the annular flow which was characterized by the continuous existence of the liquid film flow around the pipe, which as a result affected the distribution of the electrical field inside the capacitance sensor and its values.

### 4.2. Optimizing Electrodes Orientation

**Figure 14:**
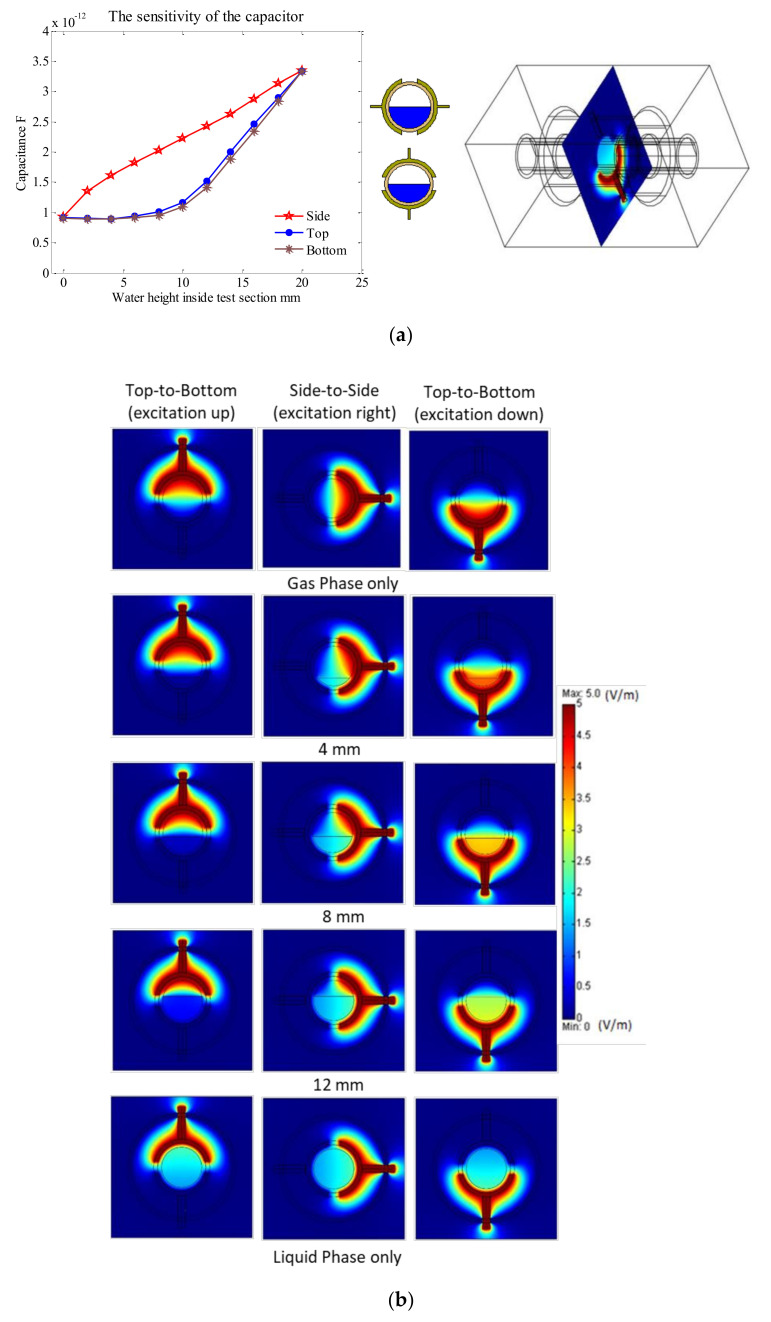
Numerically investigating the different orientations of the utilized concave two-electrode sensor around the pipe: (**a**) the capacitance values and (**b**) the electromagnetic spectrum as a function of water elevation.

After validating the numerical model against the experimental work, further analyses were conducted using the developed model. One of the important steps in testing any designed concave two-electrode capacitance sensor is to investigate the capacitance performance for different orientations of the electrodes around the test pipe (i.e., top-to-bottom and side-to-side). Initial tests were applied on the simulated sensor by increasing the elevation of water within the pipe at equal intervals which were 2 mm each for both orientations of the two electrodes. Thus, the sensitivity of the capacitance sensor was allowed to be investigated as a function of water elevation in the pipe. From Figure 14a, it can be seen that as the water level increases, the value of the measured capacitance increases. This is acceptable because as the water level increased, the corresponding electrical field distribution increased as well (Figure 14b). However, the responsiveness of the sensor was influenced by its orientation around the pipe. This influence is believed to be the artefact of the gap between the two electrodes (i.e., gap = 6 mm) and the electrical field distribution inside the model sensor. As shown in Figure 14a,b, the water phase level, its distribution around the pipe, the sensor orientation and its electrical field caused major changes in the values of the measured capacitance. There are three scenarios in terms of the excitation-electrode location for both orientations. These scenarios are top-to-bottom with excitation up or down, and side-to-side with excitation right or left (due to symmetry in the side-to-side both location being similar). From Figure 14a, the excitation for the up or down location was identical. Therefore, the top-to-bottom was not affected by the location of the excitation electrode, and accordingly, the up configuration was chosen. The side-to-side was characterized by a sharp and gradual increase, whereas the top-to-bottom was characterized by a negligible increase up until the water level reached 10 mm, and then a gradual and sharp increase was also observed. Such results, can provide that the sensor orientation is very important, however, it does not tell which orientation is best. Therefore, further numerical analysis is needed on the actual developed ideal flow patterns for both the top-to-bottom and side-to-side orientations in a multiphase flow system.

**Figure 15:**
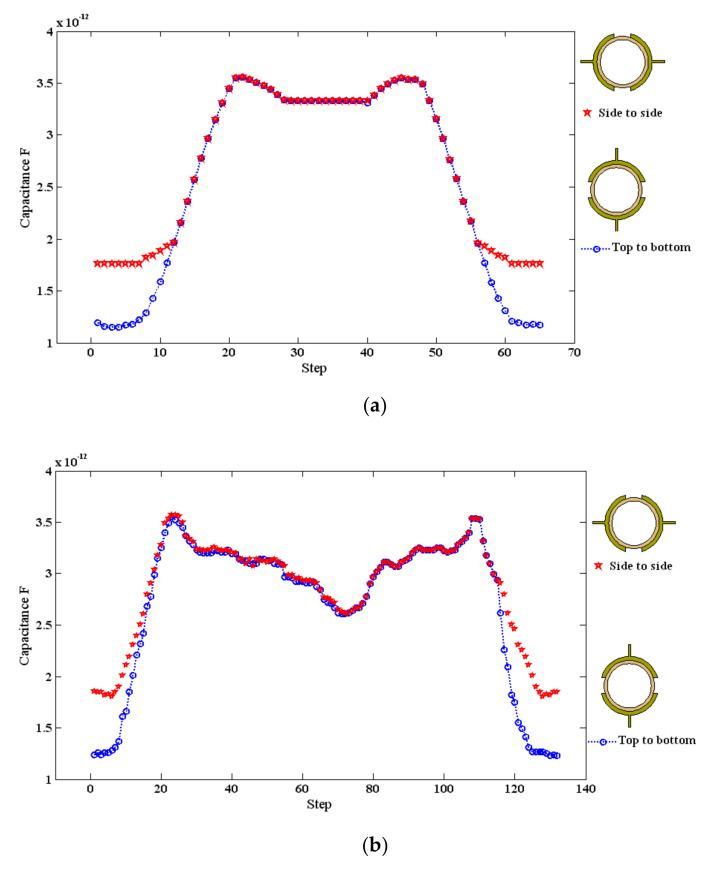

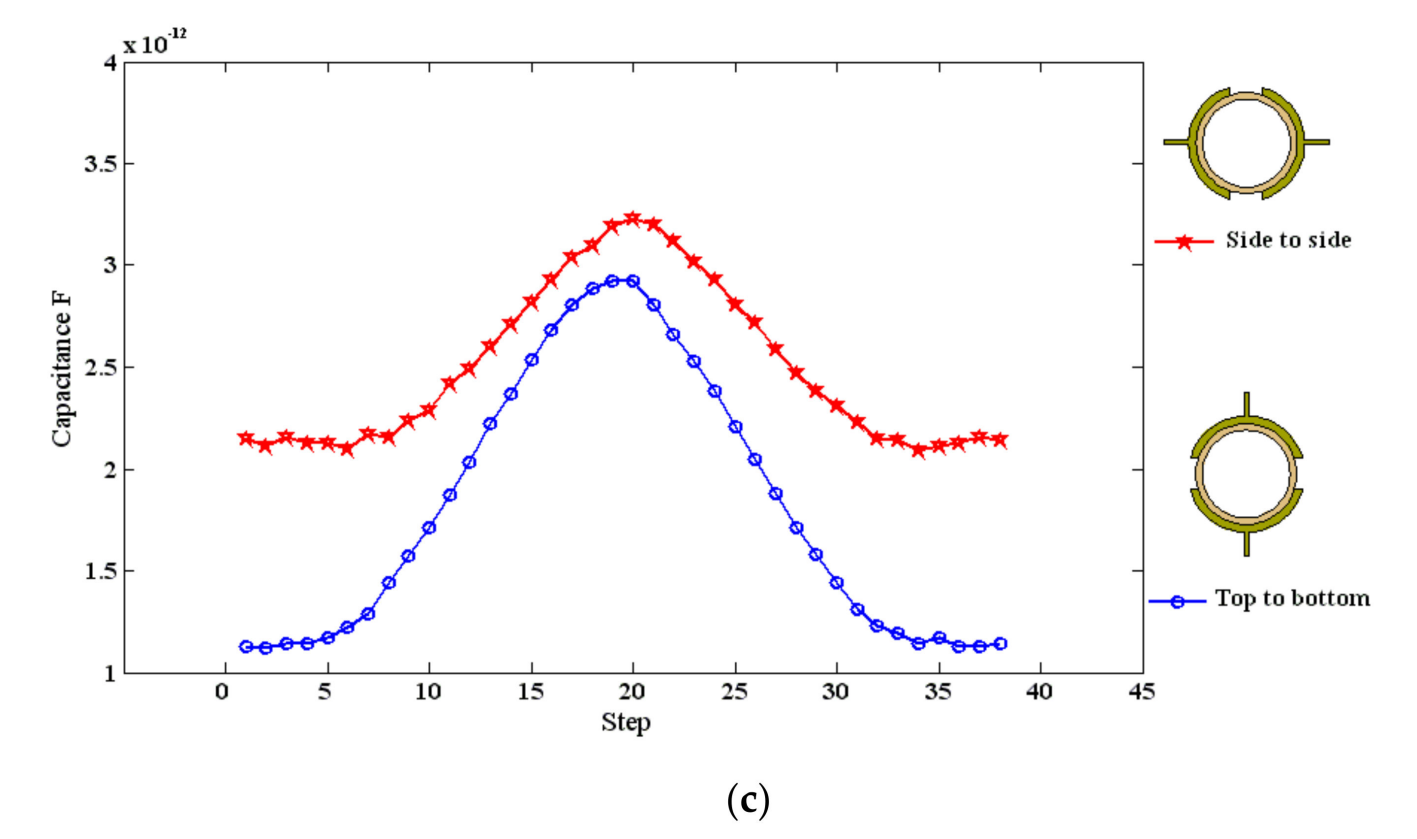
The effect of the sensor orientation on the sensor’s sensitivity and the measured capacitance values for: (**a**) the slug flow; (**b**) the slug–churn flow, and (**c**) the stratified flow (step: the advancement of the flow pattern thorough the pipe by 2 mm).

The orientation of the sensor was tested numerically for slug flow, slug–churn and stratified wavy flow patterns as shown in Figure 15a–c. This is because these flow patterns are characterized by high liquid holdup fluctuations and irregularity in the shape of the developed patterns [28,32]. Firstly, when the slug flow was tested, there is a strong difference between the capacitance value of both orientations as shown in Figure 15a. In general, the hydrodynamic classification of the slug flow can be described by the interchanging behavior of the liquid phase (i.e., the liquid slug) and the air phase almost filling the full diameter. Therefore, for the slug flow for any gas–liquid flow system at all inclinations, there was a thick film of liquid located at the lower section of the pipe. In the numerical model, this liquid film was assumed to be 2 mm. The shape of this film is a concave wedge and the top section of the liquid film ended at the middle of the tested pipe. The value of the modeled capacitance increased gradually at the first time steps, up until 10 steps when a sharp increase was observed as the liquid passed. The flow had a distinct mechanism where the highest value was 3.51 pF as its front edge reached the sensor. Later, the value declined to about 3.34 pF as the liquid filled the pipe and stayed steadily at this value until the back edge of the slug entered the capacitance domain. Due to the simulated slug edges and its geometry, again a sharp increase in the capacitance value of 3.51 pF was measured. This spike was followed by a gradual and quick decline to 1.19 pF as the air (i.e., gas phase) filled the entire volume of the pipe. Figure 15a represents the tested two orientations of electrodes around the pipe, an evident difference between the orientations can be seen. For the top-to bottom condition, the sensor sensitivity in terms of the capacitance value for the simulated case instead of being around 1.76 pF similar to the side-to-side configuration, it decreases even further to 1.19 pF. This is because at the beginning of the development of the slug flow pattern, the liquid phase occupied the lower section of the pipe. Thus, the top-to-bottom configuration was more sensitive to the existing medium than that of the side-to-side configuration.

The slug–churn pattern behavior was similar to the slug flow in terms of flow pattern structure, with the exception of that in this case the slug is considerably chaotic and bubbly, and its length was affected by the high gas flow rate. In this case, the permittivity fluctuated and the value of the capacitance fluctuated accordingly as shown in Figure 11a,b and Figure 15b. Again, the maximum capacitance values were similar for both configurations of the electrodes, except that for the side-to-side minimum capacitance value (1.90 pF) was higher than that of the top-to-bottom (1.27 pF). Once more, similar to the slug flow pattern, this is because the top-to-bottom configuration was more sensitive to the existing liquid flow at the bottom section of the pipe just before the slug–churn passed the capacitance sensor domain.

It worth noting that in Figure 15a (for the slug flow pattern) and Figure 15b (for the slug–churn flow pattern), the top to bottom and the side to side for each case had the same response at the middle portion of the figure. This section on both sides of the figures (i.e., at the beginning and the end) represents the incoming of the flow for example at step 15 (i.e., the front edge) and the outgoing of the flow at step 55 (i.e., the trailing section). At these two positions, it is very reasonable that each flow pattern for both orientations have nearly similar responses. This is because (a) high similarity in the flow pattern hydrodynamic mechanism between the slug and the slug–churn (the slug–churn is actually a liquid slug flow, but the liquid in this kind of flow is frothier, and (b) the hydrodynamic classification of the slug and slug–churn flow patterns can be described by the interchanging behavior of the liquid phase (i.e., the liquid slug) at the lower section of the pipe and high amplitude waves which would accumulate to form a region where the liquid would fill the cross-section of the pipe and be propelled forcefully by the air phase. The only difference between both figures is that when the flow was inside the capacitance sensor domain, the average permittivity of the slug–churn flow pattern was lower than that of the slug flow pattern. This manifested itself as a capacitance value of 2.6 pF for the slug–churn flow, lower than that of the slug (3.34 pF).

In addition, for the stratified flow as shown in Figure 15c, there was a clear deviation between the capacitance values depending on the electrodes’ orientation. This is believed to be due to the electrical field distribution being largely influenced by the two-phase distribution inside the pipe. This is because the stratified flow pattern is characterized by three layers of a liquid phase at the bottom section of the pipe, a gas phase at the top section of the pipe, and a wavy interface in between. As a result, the top-to-bottom orientation was more sensitive to the distribution of the existing medium at the lower half of the pipe more than that of the side-to-side orientation. For the top-to-bottom orientation, the capacitance value increased gradually by between 1.12 pF and 2.92 pF, while for the side-to-side orientation, the capacitance values increased gradually between 2.15 pF and 3.23 pF. These results disclosed that the capacitance values for the stratified flow pattern were intensely influenced by the orientation of the electrodes around the pipe.

For the small bubbles, plug, and elongated bubble flow patterns, the capacitance values for both orientations were almost similar. This because at the intermittent flow variation in the gas–liquid phases inside the pipe had little effect on the distribution of the capacitance field for both locations. The capacitance fluctuated with an average value of 3.26 pF, 3.10 pF and 3 pF for the small bubble, plug, and elongated bubble, respectively (as shown in Figure 8 and Figure 9). Thus, as can be seen from Figure 14a, above 3 pF, there is a decreasing trend and a marginal effect by the sensor’s orientation for these particular flow patterns. In Figure 14a, as the liquid phase occupied a higher volume fraction of the pipe, and the gap between the two tested orientations is becoming narrower. For these mentioned flow patterns, the liquid holdup was high and the average capacitance values were above 3 pF. Therefore, both orientations showed almost the same response for each flow pattern. Another reason for the similarity in both orientations for small bubbles, plug, and elongated bubbles is the flow pattern mechanism. There are two repeated mechanisms for these flow patterns: (a) the full flow of liquid phase, and (b) the intermittent flow of air bubbles. This behavior was repeated intermittently for all the mentioned flow patterns. The only difference is the size of the bubbles, however, the common feature between them is that the liquid occupied more than half of the pipe for all inclinations. Therefore, the capacitance for both orientations was influenced by the dominant liquid holdup more than the gas holdup.

In addition, for the annular flow pattern, the two configurations of the sensor showed similar results. However, it must be noted that not only does the holdup inside the pipe affect the values of the capacitance, but the induced shape of the flow also has a major effect. At the annular flow, the liquid film around the pipe is nonsymmetrical and fluctuates as a function of time, thus the capacitance value will fluctuate as well. Thus, the marginal difference between the two configurations is observed due to the small fluctuation in thickness. However, for the simulated case the model assumed that the layer of the liquid around the pipe is symmetrical (ideal annular flow pattern), accordingly, the capacitance will report identical values for any orientation of the electrode around the pipe.

The above results for the numerically simulated capacitance sensor demonstrated that the two orientations of the electrodes around the pipe are capable of identifying and distinguishing all kinds of two-phases flow patterns. However, when the top-to-bottom configuration was selected, the capacitance sensor was more sensitive to the developed electrical field and its distribution. In addition, it was more capable of detecting a wider range of capacitance values 1.25–1.64 times greater than that of the side-to-side’s sensitivity. It is acknowledged that this is a simplified simulation work which considered the ideal cases, cylindrical shapes and hydrodynamics assumptions. Therefore, a more sophisticated simulation is needed to (1) further understand the hydrodynamic mechanism of all the flow patterns, and (2) to optimize the design of any two-electrode capacitance sensor used in a multiphase gas–liquid system to detect the generated flow patterns. Nonetheless, the utilized numerical model discussed in this work was validated against the experimental results where good and accepted accuracy was shown.

## 5. Conclusions

This work examined the orientation sensitivity of a concave two-electrode capacitance sensor (i.e., side to side or top to bottom) which was used in a multiphase gas–liquid system. The assessment was conducted by a numerical approach using a COMSOL multi-physics simulation model. Firstly, the simulated cases were validated against comprehensive experimental data. Good agreement was found between the experimental time-dependent capacitance values when compared against the simulated capacitance values. The simulated cases captured with accepted accuracy the produced flow patterns and its variation inside the pipe. Later, the validated model was utilized to conduct further analysis on the designed sensor. In particular, the simulation was used to perform tests on the top-to-bottom and side-to-side placements of the two-electrode sensor. It was found that due to the high sensitivity of the developed electrical field and its distribution, the top-to-bottom configuration is the best orientation for the two-electrode capacitance sensor. This configuration is recommended for design purposes, performance optimization and further numerical investigations, owing to its capability in detecting a greater range of capacitance values that is 1.25–1.64 times greater than that measured by the side-to-side configuration.

## Figures and Tables

**Table 1:** A summary of the commonly utilized sensors and measurement devices in a multiphase gas–liquid flow.

Type of Sensor	Principle of Work	Usefulness
High-speed camera images ([10])	- Live recording of the generated flow pattern in the experimental work by using a viewing box to reduce the distortion due to the curvature of the test pipe.	- To identify the developed flow patterns, estimate the intermittent flow patterns ‘velocities, and measure the size of the bubble.
Photochromic dye activation ([11,12])	- A dye is used in a solution which is activated through narrow lines. These lines are crossing through a focused ultraviolet light. By recording and tracking the dye, the velocity of the used medium can be calculated by the post-processing images.	- To characterize the air bubble and the generated field around the bubble boundary.
Particles image velocimetry (PIV) ([13])	- Tracing the motion and displacement of the seeded particles inside the system.	- To measure the fluid velocity and the field corresponding to Taylor flow.
Acoustic techniques ([14])	- An external microphone sensor to identify the distinct spectrum or pulsed signal by each generated flow pattern.	- To measure the gas/liquid holdup.
Optical fiber probes ([15])	- Sensing the discrepancy in the refractive index (for the gas or liquid phase) of the generated bubbles.	- To determine the instant velocity of the bubbles inside the pipe by tracking the nose and the tails of the bubbles.
Two hot film anemometers ([16])	- Based on the heat transfer concept by cross-correlating the induced resistances of the two hot films which are proportional to the film temperature.	- To measure the bubbles’ velocity.
Conductivity and conductance probes ([17,18,19,20])	- This type of probe consists of two immersed electrodes inside the test section of a multiphase flow system, the output is estimated from the potential differences between the electrodes. Thus, the produced current is proportionate to the fluid’s conductivity that passed between the two electrodes.	- To identify the flow patterns, gas/liquid holdup and flow pattern’s transitional velocities.
Tomographic methods ([21,22,23,24,25,26])	- The average density can be calculated by detecting the X or γ rays as they were sent between the transmission point and the reflection point through the multiphase flow. - By detecting the dielectric changes induced by the two phases on the capacitance value.	- To provide visualization of the internal status of the two phases (i.e., construct cross-sectional images), gas/liquid holdup, velocity vector of the flow inside the pipe.
Static/differential pressure ports ([27])	- Obtaining the pressure between two or more points (in short or long intervals), using each pressure sensor individually to investigate the hydrodynamic fluctuations inside the test pipe by means of signal processing to find the probability density functions.	- To identify flow patterns and liquid hold.

**Table 2:** Summary of the experimental apparatus’s components, dimensions and specifications.

Component	Dimension	Specification
Test section	*l* = 4 m, *d*_in_ = 20 mm, and *d*_out_ = 24 mm	Transparent acrylic pipe
Air compressor system	Brass pipe, *l* = 2.50 m, and *d*_in_ = 9.0 mm	-
A metering system to the supply air	-	(a) Gas ball flow meter, accuracy of ±0.6%. (b) Mass flowmeter (FMA 1700/1800-Omega), accuracy of ±0.1%
Turbine flowmeter	-	FTB790-Omega, accuracy of ±0.2%
Gas–liquid mixer	*l* = 280 mm, internal pipe *d*_in_ = 20 mm	100 holes, 1 mm in diameter, dispersed 10 mm apart in the axial direction and 5 mm away from each other on the circumference
Swing table	-	Inclination range from 0° to 30°
High-speed camera images	2 min, shutter speed = 1/10,000 s, frame rate = 500/s	Installed at 3.4 m after the inlet of the test section
Tank	*V_1_* = of 0.288 m^3^ (main) *V_2_* = of 0.166 m^3^ (return)	-
Submersible pump	*-*	70 L/min
Capacitance sensor (two electrodes [32])	*d*_in_ = 24 mm, *d*_out_ = 54 mm, *l* = 54 mm, gap between electrodes = 6 mm, insulation *l* = 50 mm, and brass screen thickness = 2 mm	C_1_ at 3 m and C_2_ at 3.25 m from the inlet

**Table 3:** A summary of the experimental maps for all inclinations as a function of the two phases superficial velocities.

Flow Pattern Type	Inclination					
	Horizontal 0°		Upward 15°		Upward 30°	
	Range of Gas–Liquid Phases Superficial Velocities (m/s)					
	Gas Phase	Liquid Phase	Gas Phase	Liquid Phase	Gas Phase	Liquid Phase
Small bubbles	0.040–0.050	0.70–1.1	0.035–0.048	0.318–1.1	0.025–0.065	0.425–1.1
Plug *	N/A	N/A	0.127–0.50	0.53–1.1	0.051–0.314	0.21–1.1
Elongated bubbles	0.15–0.74	0.42–1.1	0.25–0.75	0.32–1.1	0.055–0.576	0.11–1.1
Slug	0.37–2.29	0.316–1.1	0.70–2.18	0.12–1.1	0.47–2.86	0.11–0.95
Slug–churn	2.11–3.74	0.425–1.1	2.90–4.40	0.11–1.1	2–4.29	0.10–1.1
Annular	4.48–5	0.31–1.1	4.75-5	0.106–1.1	4–5	0.11–1.1
Stratified wavy *	1.24–3	0.1–0.32	N/A	N/A	N/A	N/A

* These flow patterns did not appear: (**a**) the plug flow pattern at a horizontal 0°, and (**b**) the stratified wavy at all upward inclined angles [28,32].

**Table 4:** Schematic arrangement and explanation of the simulated flow patterns.

Type of Flow Pattern	Arrangement	Cross-Sectional View	Explanation	Number of Times Passed the Capacitance Sensor	3D Demonstration of the Defined Model Geometry
Small bubble	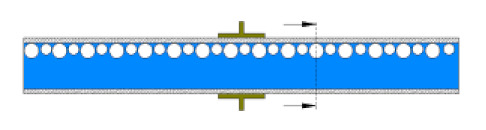	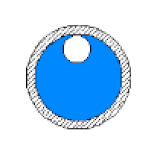	2 different sizes of small spherical bubble	100 in 10 s	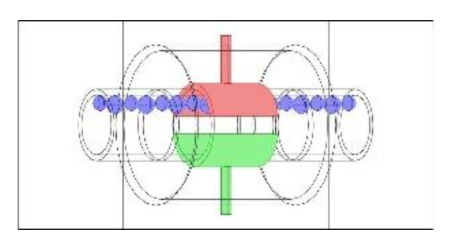
Plug	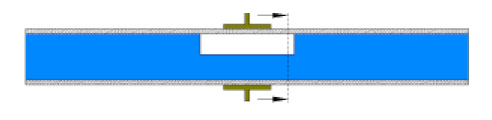	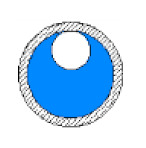	Cylindrical bubble	9 times, 9 cap bubbles in 3 s	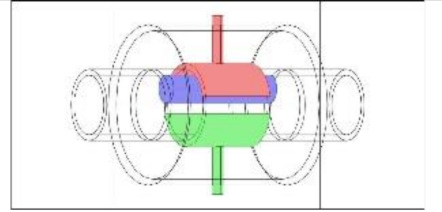
Elongated bubble	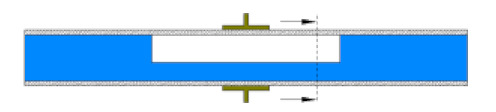	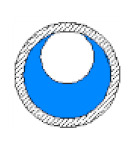	Large cylindrical bubble	15 times, 15 elongated bubbles in 4 s	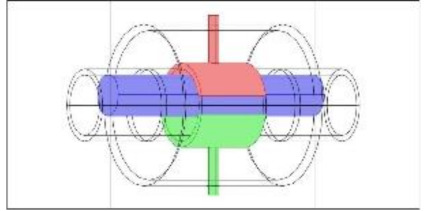
Slug	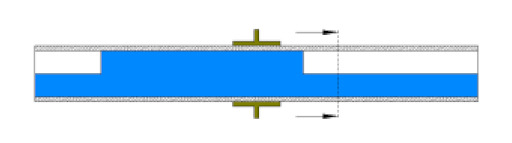	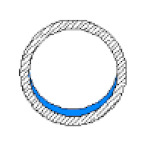	Start with a concave-shaped liquid at bottom, and later a pipe filled with liquid phase	8 times, 8 slugs in 5 s	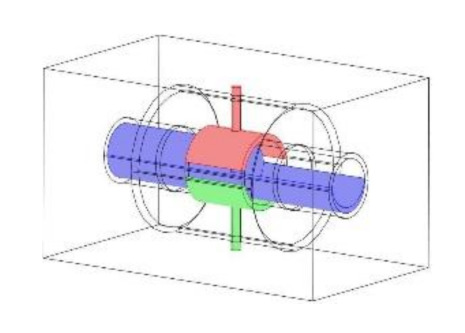
Slug–churn	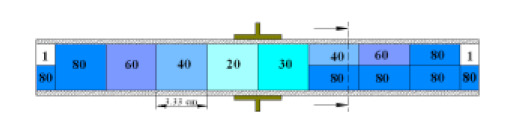	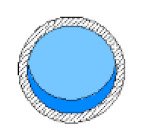	Pipe divided into 8 different permittivity sections	8 times, 8 slug–churns in 5 s	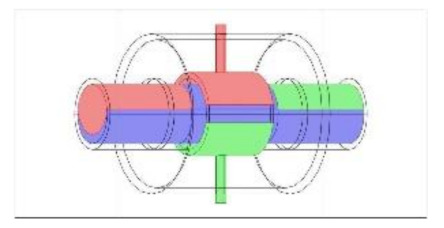
Annular	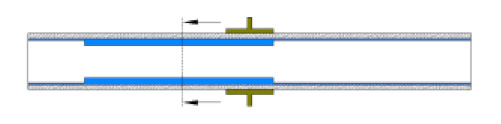	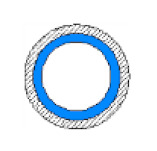	Symmetrical liquid film of thickness 1 mm, then increased by 2 mm	15 times, 15 spikes in 5 s	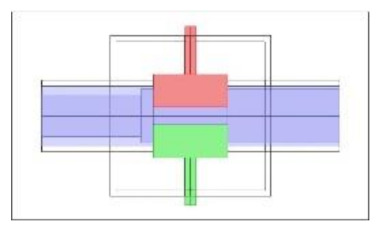
Stratified	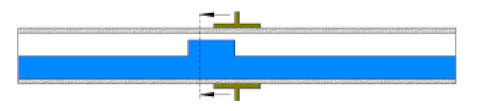	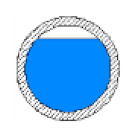	Starts with liquid up to the midline of the pipe, then a square wave of liquid crosses the sensor	8 times, 8 waves in 5 s	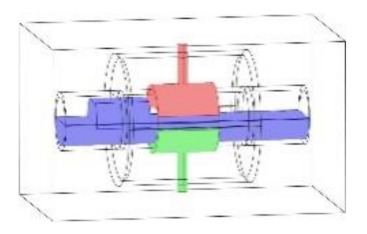

## Nomenclature

**Latin Symbols**	
C	Capacitance, pF
C_G_	Gas capacitance, pF
C_L_	Liquid capacitance, pF
C_Wall_	Wall capacitance, pF
D	Electric displacement (vector), C/m^2^
*d*_in_	Inner pipe diameter, mm
*d*_out_	Outer pipe diameter, mm
da	Infinitesimal area on closed surface, m
E	Electrical field, V/m
n	A unit vector perpendicular to da, -
*Q*	Surface charge, C
*S*	Closed surface
*u_GS_*	Superficial gas velocity, m/s
*u_LS_*	Superficial liquid velocity, m/s
*u_t_*	Translational velocity, m/s
*V(x,y,z)*	Electric potential distribution, V
*x(n)*	Discrete time signal, units depend on application
**Greek Symbols**	
$\varepsilon_{0}$	Permittivity of free space, F/m
$\varepsilon \left(x,y,z\right)$	Permittivity distribution, -
$\rho \left(x,y,z\right)$	External charge density C/m^3^
**Other Symbols**	
∇	Gradient operator
∇•	Divergence operator

## Appendix A

### Numerical Model Spikes Rationale

In order to realize whether the observed spikes were due to the flow cylindrical shape (i.e., the sharp cut) of the numerical cases, a vertical pipe with a capacitance sensor was utilized (experimentally and numerically) and was gradually filled with water, as shown in Figure A1. The capacitance values were measured at different heights of water along the vertical pipe. The water level was increased gradually until it reached the top edge of the electrodes. As can be seen in Figure A1, the capacitance values were at a minimum (i.e., 1 pF) between points the 0 and 4, representing the gas phase only [32]. As the water level increased further (in the same time maintaining the cylindrical shape of the liquid inside the pipe), a sudden and sharp increase was measured between points 4 and 5 (i.e., about 1–2 pF) and between points 5 and 6 (i.e., about 2–3.505 pF). As the water level passed the sensor ‘s screen by approximately 2 mm (point 8), the capacitance value dropped to 3.34 pF and stayed constant as the elevation of water was increased even further. This designates that the spike observed in all the time-dependent comparisons of the simulated capacitance values against the experimental work (especially for the slug flow pattern) can indeed be attributed to the cylindrical shape used in the numerical model.
